# Ag_2_CO_3_ containing magnetic nanocomposite as a powerful and recoverable catalyst for Knoevenagel condensation

**DOI:** 10.1038/s41598-021-98287-z

**Published:** 2021-09-21

**Authors:** Fatemeh Karimkhah, Dawood Elhamifar, Masoumeh Shaker

**Affiliations:** Department of Chemistry, Yasouj University, 75918-74831 Yasouj, Iran

**Keywords:** Catalyst synthesis, Heterogeneous catalysis

## Abstract

In this paper, the synthesis, characterization and catalytic application of a novel magnetic silica-supported Ag_2_CO_3_ (MS/Ag_2_CO_3_) with core–shell structure are developed. The MS/Ag_2_CO_3_ nanocomposite was prepared through chemical modification of magnetic MS nanoparticles with AgNO_3_ under alkaline conditions. The structure, chemical composition and magnetic properties of MS/Ag_2_CO_3_ were investigated by using VSM, PXRD, FT-IR, EDX and SEM techniques. The MS/Ag_2_CO_3_ nanocomposite was used as an effective catalyst for the Knoevenagel condensation under solvent-free conditions at 60 °C in an ultrasonic bath. The recovery and leaching tests were performed to study the nature of the MS/Ag_2_CO_3_ catalyst under applied conditions.

## Introduction

Magnetic nanoparticles (NPs) have wide applications in various fields of medicine, catalysis, environment, materials science and biotechnology due to their unique magnetic properties and ability to respond to external magnetic fields. Therefore, in recent years, many researchers have focused on making different types of these NPs. The cobalt, iron, nickel elements and their chemical compounds are precursors that commonly used to prepare magnetic NPs^1–9^. The use of nickel and cobalt is limited due to their toxicity and high tendency to oxidize. Among these, magnetic iron oxide NPs, especially superparamagnetic Fe_3_O_4_ NPs, have been considered by researchers due to their non-toxicity, good biocompatibility and good magnetic properties^10,11^. Magnetic NPs, despite having many advantages, suffer from a series of inherent disadvantages such as high chemical activity and a high tendency to aggregate due to their high surface area. Therefore, the development of effective strategies to improve the stability of these NPs is an essential need. Coating the surface of Fe_3_O_4_ NPs is one of the effective methods to stabilize them. The species including organic polymers such as dextran, chitosan, polyethylene glycol, polyaniline; organic surfactants such as CTAB, DTAB, DPB and SOS; metals such as Au and Ag; mineral oxides such as carbon and silica; biological molecules and structures such as ligands/receptors, peptides, liposomes have been used as coating shell for Fe_3_O_4_ NPs to form core–shell structured MNPs^10,12–24^. Some of the recently developed reports in this matter are dextran-coated Fe_3_O_4_ MNPs^12^, Fe_3_O_4_/chitosan^13^, Fe_3_O_4_/CTAB^17^, Fe_3_O_4_/Au_n_.Ac-FA NCPs^18^, Fe_3_O_4_@Ag^19^, Fe_3_O_4_@C^20^, Fe_3_O_4_-OS-SO_3_H^10^ and DOX–Fe_3_O_4_–TSL^24^. Among the various protective shells, silica has attracted more attention between many researchers. The silica shell can reduce the magnetic dipole adsorption between nanoparticles, helping to diffuse magnetic NPs in aqueous and organic environments. Also due to poor chemical permeability, silica can prevent the destruction of MNPs in different chemical environments. Moreover, the abundant silanol groups on the silica surface provide suitable conditions for different types of modification^10,25–28^. Some of recently developed magnetic nanostructures with silica shells are Fe_3_O_4_@BOS@SB/In^29^, Fe_3_O_4_@SiO_2_@PMO^30^, Re–SiO_2_–Fe_3_O_4_^31^, Mag@Ti-NOS^32^, Fe_3_O_4_@RF@void@PMO(IL)/Cu^33^, Fe_3_O_4_@SiO_2_@propyl‐ANDSA^34^ and Fe_3_O_4_@Au@mSiO_2_-dsDNA/DOX^35^. The MNPs with silica shell can be used as electrode^36^, adsorbent^37^, sensor^38^, catalyst support^10,29,32–34,39,40^, ion exchanger^41^ and so on. Especially, in the field of catalysis, magnetic silicas with a core–shell structure have been considered by many researchers due to their magnetic recoverability, high hydrophobicity and ability to modify their surface^42–46^. Some of recently developed nanocatalysts are Fe_3_O_4_@SiO_2_/Schiff-base/Cu(II)^42^, Fe_3_O_4_@SiO_2_–EDTA–Ni^43^, Fe_3_O_4_@SiO_2_-IL^44^, Fe_3_O_4_@SiO_2_/Ru-WO_x_^45^ and IL-Fe_3_O_4_@SiO_2_^46^.

Transition metal catalysts have been useful in modern synthetic organic chemistry due to their diverse reactivity in enabling various molecular conversions^47^. The reactions performed using these catalysts can be classified into three groups based on the role of the metal: 1. catalytic reactions based on the oxidation/reduction cycle of the transition metal, 2. catalytic reactions in which the transition metal acts as a Lewis acid and 3. reactions catalyzed by coinage metals (Cu, Ag and Au)^48^. In recent years, silver metal has been more considered by researchers as an effective transition metal catalyst, due to the processes catalyzed by silver perform under mild conditions and silver is cheaper and environmentally friendly than many rare metals (Pd, Pt, Rh, Ru, etc.). Among the various silver species, silver carbonate (Ag_2_CO_3_) can be employed as a Lewis acid, an inorganic base and a good oxidant in different organic reactions. Also, Ag_2_CO_3_ can be coordinated with various unsaturated systems (carbonyls, imines, isocyanides, alkynes and alkenes) and create very stable intermediates in the course of various processes^48–53^.

On the other hand, the Knoevenagel condensation of active methylene and carbonyl compounds is among the most commonly used methods in organic chemistry for the synthesis of low-electron olefins. In recent years, many catalysts were used for Knoevenagel condensation, in which heterogeneous ones have received much attention due to the easy recovery of the catalyst and also the easy separation of the products^54–61^. Some of the recently reported heterogeneous catalytic systems are Fe_3_O_4_@OS-NH_2_^54^, CAU-1-NH_2_^55^, MgO_S_400_^56^, PMO-IL-NH_2_^57^, IL–H_2_O–DABCO^58^, MgO/ZrO_2_^59^, CoFe_2_O_4_^60^ and LDH-ILs-C12^61^. In view of the above, especially the advantages mentioned for Ag_2_CO_3_, our motivation in this study is the design and preparation of a novel core–shell structured MS/Ag_2_CO_3_ nanocomposite as a powerful, effective, recyclable and reusable nanocatalyst for the Knoevenagel condensation.

## Experimental section

### Preparation of MS/Ag_2_CO_3_

For this, the Fe_3_O_4_ NPs (0.6 g)^29^ were dispersed in deionized water (25 mL) and EtOH (75 mL) for 0.5 h. After adding NH_3_ (3.5 mL, 25% wt), the mixture was stirred at RT for 20 min. Then, tetramethoxysilane (TMOS, 0.5 mL) was added and stirring was continued at RT for 16 h. After that, the resulting solid material was magnetically collected, washed with deionized water and EtOH, dried at 80 °C for 6 h and defined as MS. For preparation of MS/Ag_2_CO_3_, MS (0.6 g) was well-dispersed in deionized water (30 mL). After 0.5 h, NaHCO_3_ (2.5 mmol) was added and stirring was continued at RT for 2 h. Then, AgNO_3_ (5 mmol) was added under lightless conditions. After that, the reaction combination was stirred for 12 h in an ice bath. The resulted material was collected using a magnetic field, washed with deionized water, dried and designated as MS/Ag_2_CO_3_.

### Procedure for the Knoevenagel reaction using MS/Ag_2_CO_3_

For this, MS/Ag_2_CO_3_ (0.015 g), ethyl cyanoacetate (1 mmol) and aldehyde (1 mmol) were added in a reaction flask and the resulted mixture was sonicated at 60 °C under solvent-free conditions. After completing of the process, EtOH (10 mL) was added and MS/Ag_2_CO_3_ was magnetically separated. Finally, the solvent was evaporated and pure Knoevenagel products were resulted after recrystallization in EtOH and n-hexane solvents.

### IR, ^1^H NMR and ^13^C NMR data of Knoevenagel products

#### (E)-ethyl 2-cyano-3-(2-nitrophenyl)acrylate (Table 2, entry 2)

Pale yellow solid; yield: 95%; M.P.: 98–100 °C (ref: 102 °C^62^), IR (KBr, cm^−1^): 3097 (=C–H, stretching vibration sp^2^), 2989 (C–H, stretching vibration sp^3^), 2221 (CN, stretching vibration), 1723 (C=O, stretching vibration), 1565, 1462 (C=C, Ar stretching sp^2^), 1264 (C–O, stretching vibration), 1529, 1358 (NO_2_, stretching vibration). ^1^H NMR (300 MHz, DMSO): δ (ppm) 1.34 (t, 3H, *J* = 6.0 Hz), 4.38 (q, 2H), 7.84–7.89 (m, 1H), 7.93–8.02 (m, 2H), 8.33 (d, 1H, *J* = 9.0 Hz), 8.86 (s, 1H). ^13^C NMR (75 MHz, DMSO): δ (ppm) 14.4, 63.1, 107.8, 114.7, 125.7, 128.7, 131.0, 132.9, 135.2, 147.7, 155.6, 161.4.

#### (E)-ethyl 2-cyano-3-(4-nitrophenyl)acrylate (Table 2, entry 3)

Pale yellow solid; yield: 97%; M. P.: 170–171 °C (ref: 168 °C^63^), IR (KBr, cm^−1^): 3095 (=C–H, stretching vibration sp^2^), 2990 (C–H, stretching vibration sp^3^), 2226 (CN, stretching vibration), 1718 (C=O, stretching vibration), 1593, 1469 (C=C, Ar stretching sp^2^), 1259 (C–O, stretching vibration), 1510, 1350 (NO_2_, stretching vibration). ^1^H NMR (300 MHz, DMSO): δ (ppm) 1.31 (t, 3H, *J* = 6.9 Hz), 4.33 (q, 2H), 7.82 (d, 2H, *J* = 13.2 Hz), 8.00 (d, 2H, *J* = 10.80 Hz), 8.40 (s, 1H). ^13^C NMR (75 MHz, DMSO): δ (ppm) 14.4, 62.9, 103.7, 115.8, 127.7, 131.0, 132.9, 133.0, 154.3, 162.1.

#### (E)-ethyl 3-(2-chlorophenyl)-2-cyanoacrylate (Table 2, entry 4)

White solid; yield: 92%; M.P.: 52–54 °C (ref: 52–54 °C^64^), IR (KBr, cm^−1^): 3072 (=C–H, stretching vibration sp^2^), 2955 (C–H, stretching vibration sp^3^), 2229 (CN, stretching vibration), 1718 (C=O, stretching vibration), 1619, 1475 (C=C, Ar stretching sp^2^), 1264 (C–O, stretching vibration). ^1^H NMR (400 MHz, CDCl_3_): δ (ppm) 1.43 (t, 3H, *J* = 7.2 Hz), 4.43 (q, 2H), 7.40–7.47 (m, 1H), 7.50–7.55 (m, 2H), 8.24 (d of d, 1H, *J*_*1*_ = 4.6 Hz, *J*_*2*_ = 1.6 Hz,), 8.71 (s,1H). ^13^C NMR (100 MHz, CDCl_3_): δ (ppm) 14.2, 62.9, 106.2, 114.8, 127.5, 129.8, 129.9, 130.3, 133.7, 136.4, 151.1, 161.8.

#### (E)-ethyl 2-cyano-3-(p-tolyl)acrylate (Table 2, entry 7)

White solid; yield: 91%; M.P.: 95–97 °C (ref: 93–94 °C^62^), IR (KBr, cm^−1^): 3025 (=C–H, stretching vibration sp^2^), 2961 (C-H, stretching vibration sp^3^), 2217 (CN, stretching vibration), 1725 (C=O, stretching vibration), 1604, 1515 (C=C, Ar stretching sp^2^), 1261 (C–O, stretching vibration). ^1^H NMR (400 MHz, CDCl_3_): δ (ppm) 1.42 (t, 3H, *J* = 7.2 Hz), 2.46 (s, 3H), 4.42 (q, 2H), 7.33 (d, 2H, *J* = 8.4 Hz), 7.93 (d, 2H, *J* = 8.4 Hz), 8.25 (s, 1H). ^13^C NMR (100 MHz, CDCl_3_): δ (ppm) 14.2, 22.0, 62.6, 101.5, 115.8, 128.8, 130.1, 131.3, 144.7, 155.0, 162.8.

## Results and discussion

**Figure 1:**
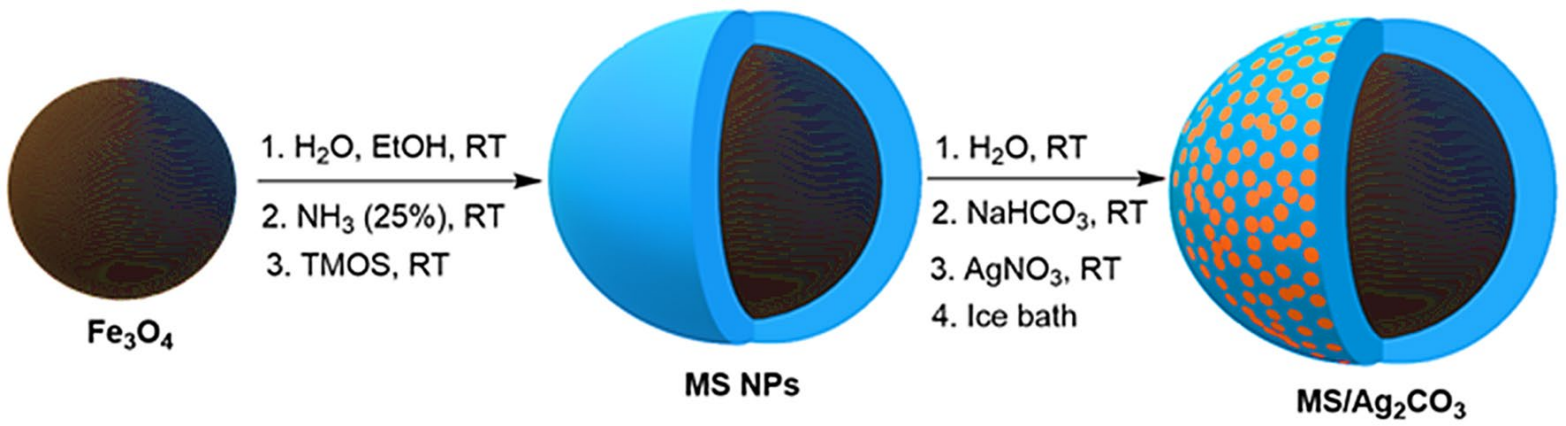
Preparation of the MS/Ag_2_CO_3_ nanocomposite.

The synthesis of MS/Ag_2_CO_3_ is shown in Fig. 1. Firstly, the Fe_3_O_4_ NPs were modified with a silica shell to give MS NPs. Then, the MS NPs were treated with NaHCO_3_ and AgNO_3_ to deliver the desired MS/Ag_2_CO_3_ nanocomposite.

**Figure 2:**
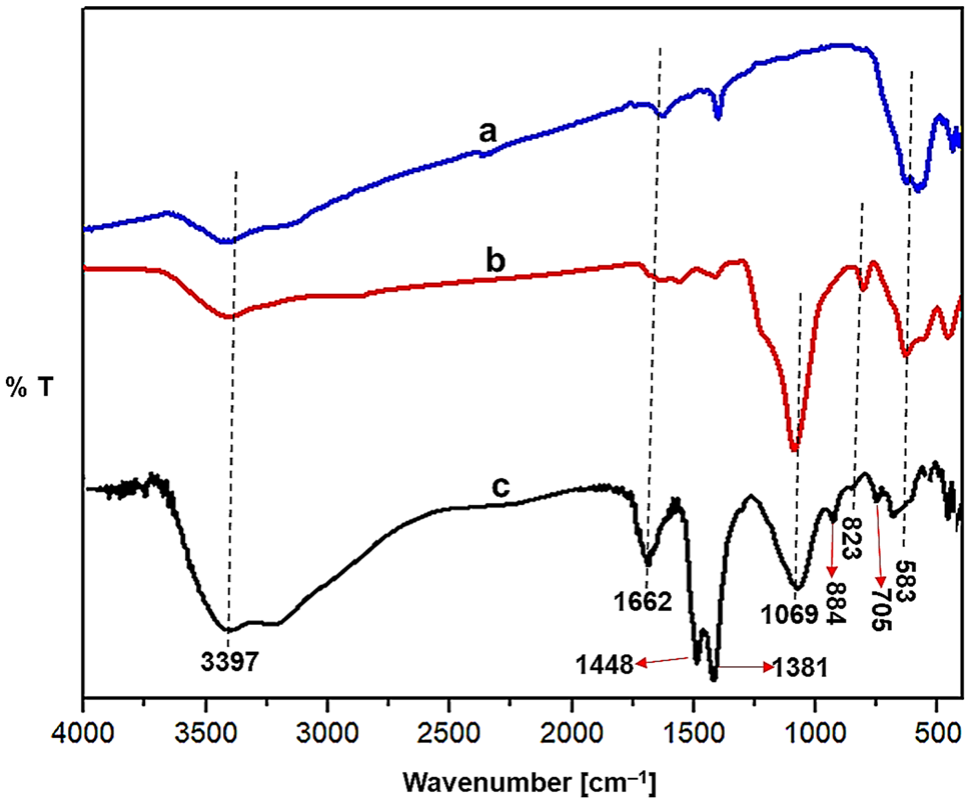
FT-IR of (**a**) Fe_3_O_4_, (**b**) MS and (**c**) MS/Ag_2_CO_3_.

The FT-IR spectra of Fe_3_O_4_, MS and MS/Ag_2_CO_3_ are shown in Fig. 2. For all samples, the characteristic signals at 3397 and 583 cm^−1^ are, respectively, due to O–H and Fe–O bonds. Also, the band cleared at 1662 cm^−1^ is due to bending vibration of O–H bonds^32,65^. For MS and MS/Ag_2_CO_3_, the signals at 823 and 1069 cm^−1^ are assigned to Si–O-Si, confirming the construction of SiO_2_ shell around the Fe_3_O_4_ core (Fig. 2b,c)^26^. Importantly, for the MS/Ag_2_CO_3_ nanocomposite, the observed peaks at 705, 884, 1381 and 1448 cm^−1^ are attributed to the absorption bands of CO_3_^2^ (Fig. 2c), indicating successful immobilization of Ag_2_CO_3_ particles on the surface of MS^66^.

**Figure 3:**
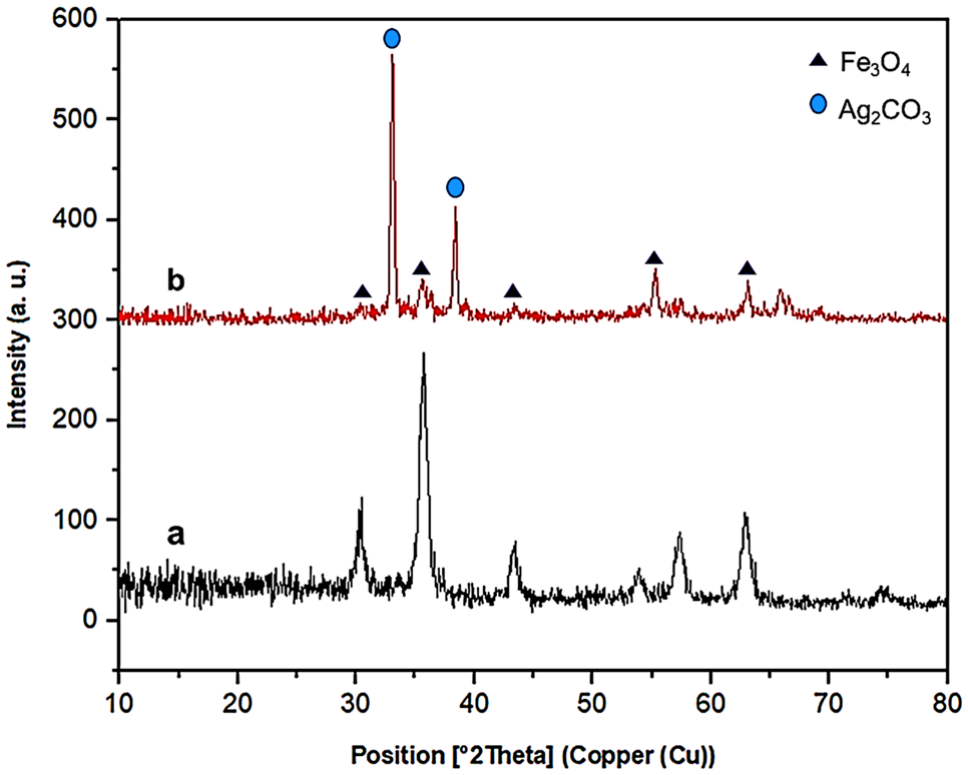
Wide angle-PXRD pattern of the (**a**) Fe_3_O_4_ and (**b**) MS/Ag_2_CO_3_ nanomaterials.

The wide-angle PXRD pattern of Fe_3_O_4_ and MS/Ag_2_CO_3_ nanomaterials are shown in Fig. 3. As shown, for both samples, six characteristic peaks are observed at 2θ of 30.10, 35.58, 43.29, 53.81, 57.44 and 63.24 degree, corresponding to the crystal planes of (220), (311), (400), (422), (511) and (440), respectively. These are related to the crystalline structure of magnetite NPs confirming high stability of Fe_3_O_4_ during catalyst preparation. The pattern of MS/Ag_2_CO_3_ nanocomposite also showed two sharp peaks at 2θ of 33.2 and 38.5 degree corresponding to the Ag_2_CO_3_ NPs (Fig. 3b)^10,66,67^. This proves successful construction of Ag_2_CO_3_ NPs on MS core.

**Figure 4:**
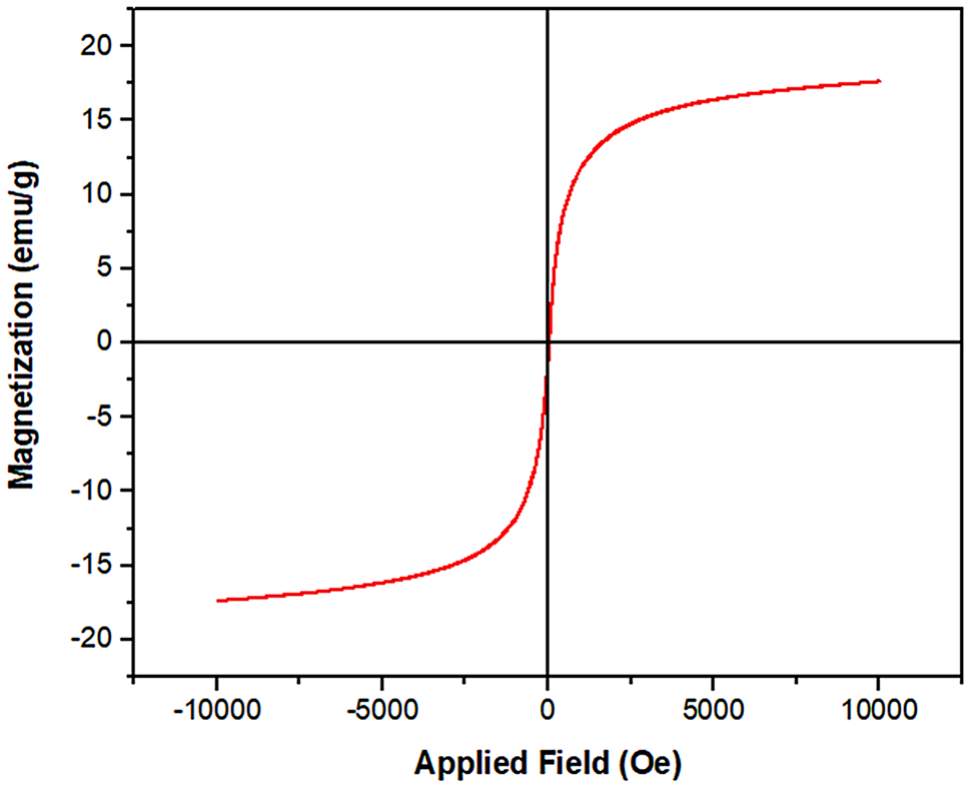
VSM of the MS/Ag_2_CO_3_.

The VSM analysis was done to investigate the magnetic property of MS/Ag_2_CO_3_ nanocomposite (Fig. 4). As shown, the saturation magnetization of 17.5 emu/g was found for this material. Also, the VSM curve showed that this material has a superparamagnetic behavior.

**Figure 5:**
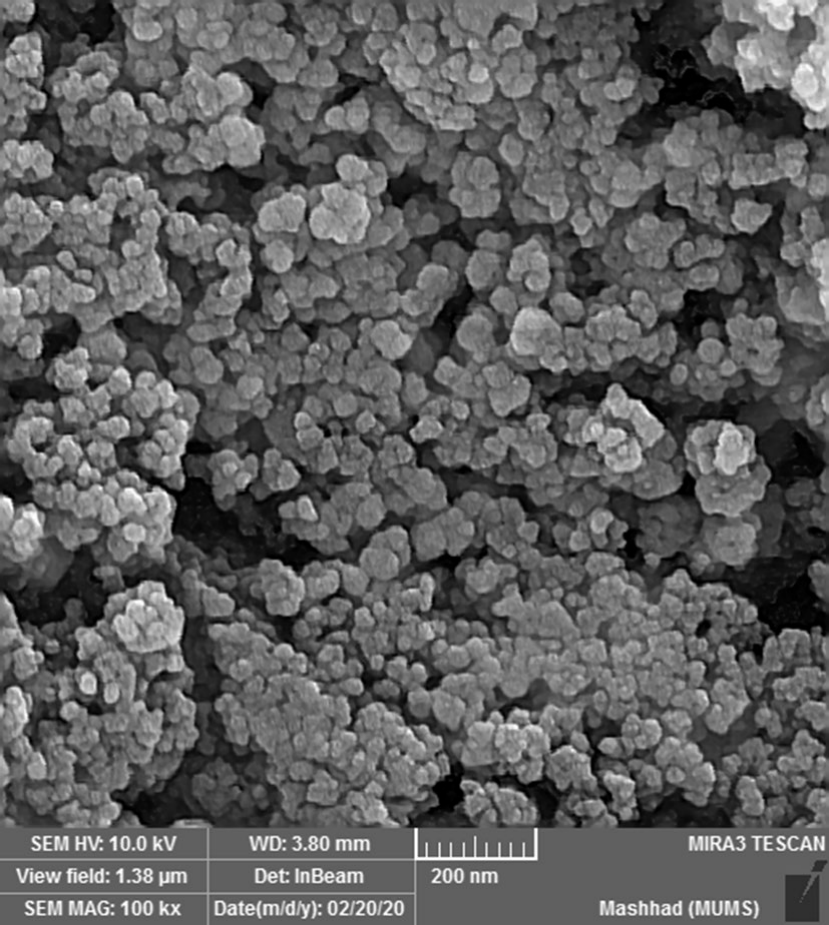
SEM image of MS/Ag_2_CO_3_.

The surface morphology of MS/Ag_2_CO_3_ nanocomposite was studied by using SEM analysis. This showed that the MS/Ag_2_CO_3_ nanocomposite has a uniform spherical structure (Fig. 5).

**Figure 6:**
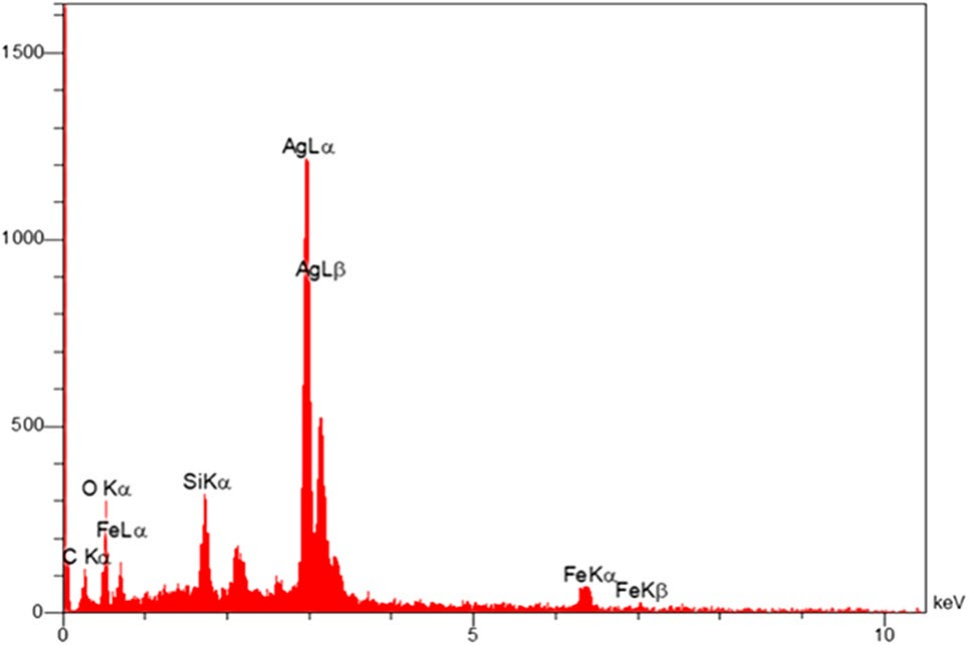
EDX of MS/Ag_2_CO_3_ nanocomposite.

The EDX spectrum showed that the designed MS/Ag_2_CO_3_ is composed of Fe, Si, O, Ag and C elements confirming the successful incorporation/immobilization of expected species in the material framework (Fig. 6).

In the following, the catalytic activity of MS/Ag_2_CO_3_ was evaluated in the Knoevenagel reaction. To obtain the optimum conditions, the reaction of benzaldehyde with ethyl cyanoacetate was chosen as a model (Table 1). The study showed that the catalyst loading is a very important factor in the reaction progress, in which the best result was obtained using 0.015 g of catalyst (Table 1, entries 1–4). The solvent screening demonstrated that solvent-free condition is the best for the reaction (Table 1, entries 4–8). Evaluation of temperature showed that the highest activity of MS/Ag_2_CO_3_ is resulted at 60 °C (Table 1, entry 4 *versus* entries 9, 10). In the next, the activity of Ag_2_CO_3_-free Fe_3_O_4_ and MS nanomaterials was compared with that of MS/Ag_2_CO_3_ showing that the presence of Ag_2_CO_3_ particles as active catalytic centers are necessary for the development of the reaction (Table 1, entry 4 *versus* entries 11, 12). To prove the effect of both Ag and CO_3_ species in the reaction progress, the catalytic activity of MS/Ag_2_CO_3_ was compared with AgCl, Na_2_CO_3_ and NaNO_3_ salts (Table 1, entry 4 *versus* entries 13–15). The results showed that AgCl and Na_2_CO_3_ deliver a low to moderate yield of desired product. While, using NaNO_3,_ no progress was observed in the reaction. Interestingly, in the presence of designed MS/Ag_2_CO_3_ the best result was obtained. These confirm that both Ag and CO_3_ species are necessary for the development of reaction. According to these results, it can be concluded that the designed MS/Ag_2_CO_3_ acts as a bifunctional catalyst in the reaction.

**Table 1:** The effect of catalyst, solvent and temperature in the Knoevenagel condensation.


Entry	Catalyst	Cat. (g)	Solvent	T (°C)	Yield (%)^a^
1	–	–	Solvent-free	60	–
2	MS/Ag_2_CO_3_	0.005	Solvent-free	60	78
3	MS/Ag_2_CO_3_	0.01	Solvent-free	60	85
**4**	**MS/Ag**_**2**_**CO**_**3**_	**0.015**	**Solvent-free**	**60**	**96**
5	MS/Ag_2_CO_3_	0.015	Toluene	60	68
6	MS/Ag_2_CO_3_	0.015	EtOH/Toluene (50:50)	60	75
7	MS/Ag_2_CO_3_	0.015	EtOH	60	82
8	MS/Ag_2_CO_3_	0.015	H_2_O	60	84
9	MS/Ag_2_CO_3_	0.015	Solvent-free	45	81
10	MS/Ag_2_CO_3_	0.015	Solvent-free	75	96
11	Fe_3_O_4_	0.015	Solvent-free	60	34
12	MS	0.015	Solvent-free	60	–
13	Na_2_CO_3_	0.015	Solvent-free	60	43
14	AgCl	0.015	Solvent-free	60	27
15	NaNO_3_	0.015	Solvent-free	60	–

^a^Reaction condition: benzaldehyde (1 mmol) and ethyl cyanoacetate (1 mmol) under ultrasonic waves for 20 min.

^b^Isolated yields.

With the optimal conditions in hand that are bolded in Table 1 (entry 4), a variety of aldehydes were employed as substrate (Table 2). Generally, for aromatic aldehydes bearing both electron-withdrawing or electron-donating substituents, electronic nature or substitution pattern had little effect on this process and MS/Ag_2_CO_3_ was able to effectively catalyze the reaction to give the Knoevenagel products in high to excellent yields. Also, terephthalaldehyde, hetero-aromatic aldehydes such as thiophene-2-carbaldehyde and furan-2-carbaldehyde, 1-naphthaldehyde and hexanal also gave the corresponding Knoevenagel adducts in good to high yield at relatively short time. It is important to note that, as previously summarized by Tietze et al., all condensations of cyanoacetate with aromatic and aliphatic aldehydes give E-isomer almost exclusively^68^. In the present study, all synthesized Knoevenagel products were identified as E-isomer by comparing their melting points, IR, and NMR spectra with valid samples^62–64,69–72^.

**Table 2:** Preparation of the Knoevenagel products using MS/Ag_2_CO_3_.


Entry	Aldehyde	Time (min)	Yield (%)^a^	Found M. P. (°C)	Reported M. P. (°C)
1		20	96	51–53	50–51^62^
2		20	95	98–100	102^62^
3		15	97	170–171	168^63^
4		25	92	52–54	52–54^64^
5		18	94	87–89	87–89^62^
6		15	96	88–90	90–91^70^
7		25	91	95–96	93–94^62^
8		30	88	170–171	169^62^
9^b^		20	91	198–200	200^71^
10		20	93	85–87	85–87^64^
11		22	92	95–97	95–96^72^
12		20	94	82–84	81–82^71^
13		30	83	Colorless oil	Colorless oil^64^

^a^Isolated yields.

^b^Ethyl cyanoacetate (2 mmol).

**Figure 7:**
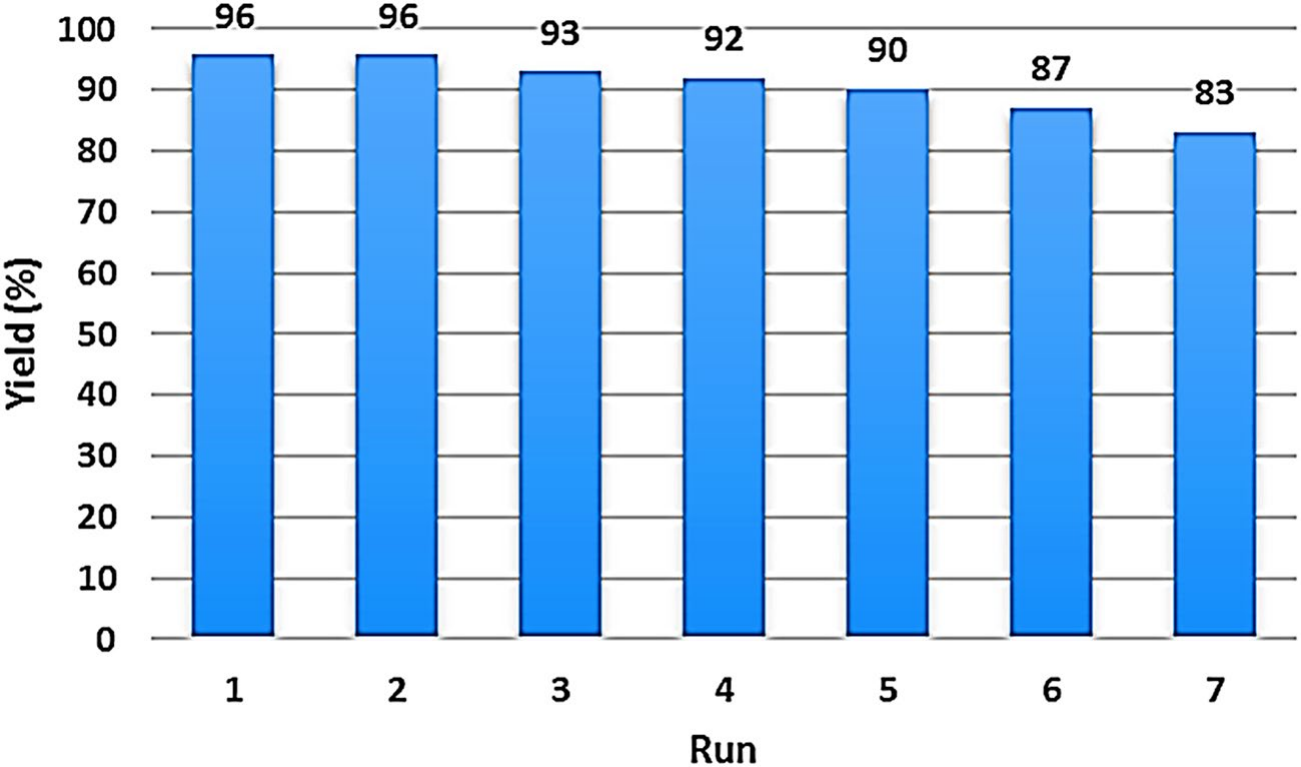
Reusability of the MS/Ag_2_CO_3_ nanocatalyst.

The ability to recycle and reuse of catalysts are important issues that should be considered in heterogeneous catalytic systems. In this regard, the recyclability and reusability of MS/Ag_2_CO_3_ catalyst were evaluated in the condensation of benzaldehyde with ethyl cyanoacetate as a test model. For this, in the end of reaction, the catalyst was magnetically separated and reused in the next reaction run at conditions the same as first run. As it is clear in Fig. 7, MS/Ag_2_CO_3_ can be recovered and reused at least 6 times without the significant loss in its activity and productivity.

For determining the nature of the MS/Ag_2_CO_3_ catalyst, a leaching test was done at optimal conditions. For this, after progress of about 50% of the process, the MS/Ag_2_CO_3_ catalyst was collected using a magnet and the reaction progress of the catalyst-free residue was monitored. Importantly, after 1 h, no progress was observed in converting the starting material to product. This proves the heterogeneous nature of the MS/Ag_2_CO_3_ catalyst and also confirms no-leaching of active Ag_2_CO_3_ particles during the applied conditions.

**Figure 8:**
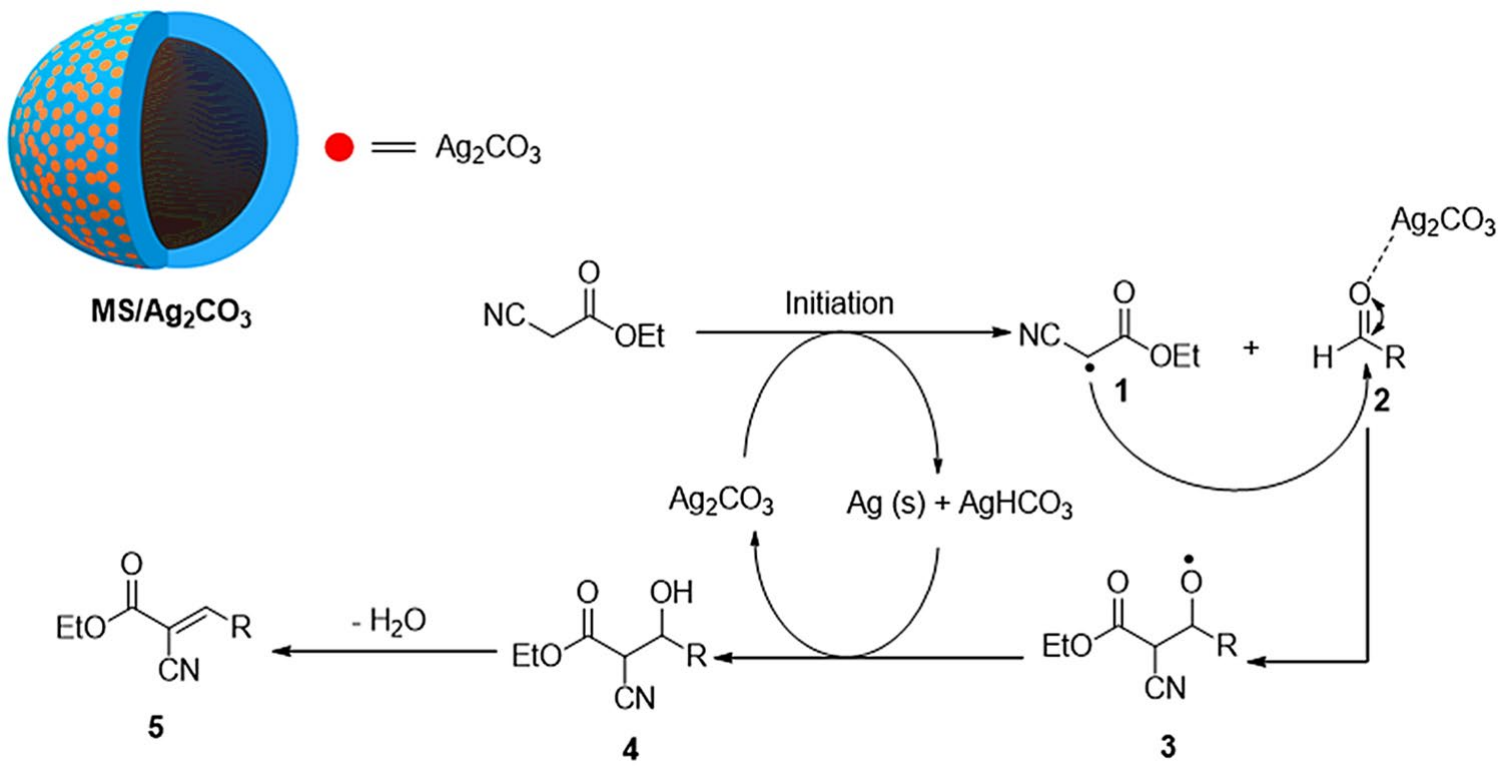
Proposed mechanism for the Knoevenagel condensation using the MS/Ag_2_CO_3_ catalyst.

Although the exact reaction pathway for the Knoevenagel condensation with the MS/Ag_2_CO_3_ catalyst is not clear for us, however, based on the results presented for the Ag_2_CO_3_ catalyst in other organic reactions, a plausible mechanism for this reaction is presented in Fig. 8. Since Ag_2_CO_3_ has a dual role as a base and a one-electron oxidant^48^, it picks up one acidic proton from the active methylene group of ethyl cyanoacetate to give radical intermediate **1**. Simultaneously, Ag_2_CO_3_ coordinates to an aldehyde to generate complex **2**. Then, the intermediate **1** is coupled with complex **2** to give radical intermediate **3**. In the next step, radical intermediate **3** provides β-hydroxyl compound **4** by picking up a H atom from the produced AgHCO_3_ during the one-electron oxidation. Finally, the desired Knoevenagel product **5** is resulted after dehydration of the β-hydroxyl compound.

At the end, the performance of the MS/Ag_2_CO_3_ catalyst was compared with the previous catalysts in the Knoevenagel condensation (Table 3). As demonstrated, the study showed that MS/Ag_2_CO_3_ is a catalyst with higher efficiency, stability and durability time than other catalysts. These findings are attributed to the magnetic nature and the chemically immobilized Ag_2_CO_3_ particles. In fact, the high performance of Ag_2_CO_3_ NPs in the catalytic processes is due to its bifunctional role as both inorganic base and Lewis acid.

**Table 3:** Comparison of the catalytic activity of MS/Ag_2_CO_3_ with other catalysts.

Catalyst	Conditions	Recovery times	Refs
FeNPs/PPD@rGO	Cat. 0.05 g, Toluene, 40 °C, 3.5 h	6	^73^
Y_2_ZnO_4_	Cat. 0.0136 g, solvent-free, under MW (420 W), 15 min	3	^74^
CoFe_2_O_4_	Cat. 0.01 g, water/ethanol, 50 °C, 20 min	4	^60^
Zn-MOF	Cat. 0.0693 g, toluene, 50 °C, 48 h	4	^75^
Mg-ABDC	Cat. 0.045 g, ethanol, 80 °C, 7 h	5	^76^
Mg@PS-anthra	Cat. 0.05 g, solvent-free, r. t., 2 h	5	^77^
MS/Ag_2_CO_3_	Cat. 0.015 g, solvent-free, 60 °C, 20 min, )))	6	This work

*PPD* p-phenylenediamine, *rGO* reduced graphene oxide, *MOF* metal–organic framework, *ABDC* 2-aminobenzene-1,4-dicarboxylate anion, *PS* polystyrene, *anthra* anthranilic acid.

## Conclusion

In summary, a novel magnetic silica-supported Ag_2_CO_3_ (MS/Ag_2_CO_3_) was successfully prepared and its catalytic performance was studied. The FT-IR and EDX techniques showed the well immobilization of Ag_2_CO_3_ particles on the MS nanomaterial. The wide-angle PXRD analysis demonstrated the high stability of Fe_3_O_4_ NPs during steps of catalyst preparation. The PXRD pattern also confirmed the well formation of Ag_2_CO_3_ NPs on the MS nanocomposite. The superparamagnetic behavior of MS/Ag_2_CO_3_ was confirmed by the VSM analysis. The SEM image also demonstrated a uniform spherical structure for this catalyst. The MS/Ag_2_CO_3_ nanocatalyst was efficiently employed in the Knoevenagel condensation under moderate conditions and delivered the desired products in high to excellent yield. Also, MS/Ag_2_CO_3_ could be recycled and reused with maintaining its activity in several times.
